# Microperimetry as an Outcome Measure in *RPGR-*associated Retinitis Pigmentosa Clinical Trials

**DOI:** 10.1167/tvst.12.6.4

**Published:** 2023-06-09

**Authors:** Laura J. Taylor, Amandeep S. Josan, Jasleen K. Jolly, Robert E. MacLaren

**Affiliations:** 1 Nuffield Laboratory of Ophthalmology, Nuffield Department of Clinical Neurosciences, University of Oxford, Oxford, UK; 2 Oxford Eye Hospital, Oxford University Hospitals NHS Foundation Trust, Oxford, UK; 3 Vision and Eye Research Institute, Anglia Ruskin University, Cambridge, UK

**Keywords:** microperimetry, retinitis pigmentosa, outcome measure, clinical trial, volume sensitivity

## Abstract

**Purpose:**

To explore which microperimetry sensitivity index (pointwise sensitivity, mean sensitivity, and volume sensitivity) is suitable as a microperimetry outcome measure in patients with X-linked *RPGR-*associated retinitis pigmentosa (RP).

**Methods:**

Microperimetry data from patients with *RPGR*-associated RP were collected and analyzed retrospectively. Fourteen participants completed triplicate microperimetry testing, across 2 consecutive days for the repeatability analyses. Longitudinal data was obtained from 13 participants who completed microperimetry testing at two separate visits.

**Results:**

The test–retest coefficients of repeatability (CoR) for pointwise sensitivity were ±9.5 dB and ±9.3 dB, in the right and left eyes, respectively. The mean sensitivity CoR for the right and left eyes was ±0.7 dB and ±1.3 dB. Volume sensitivity CoR was ±144.5 dB*deg^2^ and ±324.2 dB*deg^2^ for the right and left eyes, respectively. The mean sensitivities were positively skewed toward zero in those with a high number of nonseeing points (arbitrarily assigned to −1.0 dB) and just seen points (0.0 dB). Volume sensitivities were unaffected by the averaging effects of skewed data.

**Conclusions:**

Clinical trials should report population-specific test–retest variability to determine a clinically significant change. Pointwise sensitivity indices should be used with caution as outcome measures in clinical trials owing to high levels of test–retest variability. Global indices seem to be less prone to variability. Volume sensitivity indices seem to be superior for use in *RPGR*-associated RP clinical trials compared with mean sensitivity because they are unaffected by the averaging effects of highly skewed data.

**Translational Relevance:**

Careful selection of sensitivity indices (VA) is required when using microperimetry as a clinical trial outcome measure.

## Introduction

*RPGR*-associated retinitis pigmentosa (RP) is one of the most common and severe causes of RP worldwide.^1^ Currently, there is no treatment for *RPGR*-associated RP, but gene therapy clinical trials have been underway for the last 5 years.^2^^,^^3^ *RPGR*-associated RP typically (although not always) presents as a rod-cone dystrophy and is characterized by early onset nyctalopia, followed by progressive peripheral visual field loss. Visual acuity (VA) is moderately preserved until the retinal degeneration encroaches centrally, occurring around the fourth decade and resulting in severe sight impairment.^4^ Consequently, VA is considered an ineffective marker of visual function and disease progression in early to middle stages, thus making it a suboptimal outcome measure for monitoring effects of novel therapeutic interventions. Hence, alternative visual function markers are required.

The ideal outcome measure would detect visual function changes to novel therapeutic interventions, without being affected by natural patient variability. Microperimetry, also known as fundus-controlled perimetry, has become a popular device for use as an outcome measure in clinical trials for inherited retinal disease including *RPGR*-associated RP.^5^^,^^6^ The Icare Macular Integrity Assessment (MAIA) confocal microperimeter (Mainline Instruments Limited, Kings Norton, Birmingham, UK) combines a scanning laser ophthalmoscope, real-time fundus tracking, and perimetry to accurately assess central retinal sensitivity. The posterior fundus is visualized via an infrared super luminescent diode, and fundus landmark features are registered and tracked in real time. Stimuli positions are altered dynamically to compensate for any fixational movements before being presented.^7^ Retinal sensitivity at a single locus is reported via a point threshold (often termed pointwise sensitivity) measured on a decibel (dB) logarithmic scale. From this information, an average of all pointwise sensitivity values provides an overall mean sensitivity. However, the averaging of any highly skewed group of pointwise sensitivities (away from a normal distribution) may not represent the overall sensitivity accurately. Small and focal sensitivity changes often go undetected due to averaging effects. Volume sensitivity is the product of points or region of sensitivities multiplied by the area covered by that point or region.^8^^,^^9^ Therefore, volume sensitivity does not rely on conditions of data distribution normality and so is unaffected by averaging effects from highly skewed distributions of pointwise sensitivities or unequal grid spacing, such as the often used radial grid patterns.^8^

Optimum outcome measures provide reliable evidence for regulatory approval, including the U.S. Food and Drug Administration (FDA) and the UK Medicines and Healthcare products Regulatory Agency (MHRA). These outcome measures must show clinically meaningful changes via predefined measurable clinical endpoints and not simply show statistical significance. The FDA previously suggested for clinical trials using standard automated perimetry, such as in glaucoma trials, a between-group mean difference (between treated and untreated control cohorts) of at least a 7.0 dB mean sensitivity change, for the entire field, could be considered clinically significant.^10^ Subsequently, two recent gene therapy trials for *RPGR-*associated RP have applied a similar criteria as an endpoint for clinical significance, reporting the number of individuals with at least 5 points showing a sensitivity gain of ≥7.0 dB in the treated eye, within the central 16 and 36 loci (when using the 10-2, 68-point testing grid).^11^^,^^12^ Meanwhile, two active *RPGR*-associated RP clinical trials simply state a change from baseline microperimetry sensitivity as secondary outcome measures without providing more specific endpoint criteria (NCT 03584165 and NCT 03349242). Despite the popularity of microperimetry as an outcome measure in *RPGR*-associated RP clinical trials, specific endpoint criteria suitability is yet to be explored.

This study aims to analyze microperimetry sensitivity data from patients with *RPGR*-associated RP, to identify which microperimetry sensitivity index is most suitable to be used as a microperimetry outcome measure and endpoint in future *RPGR*-associated RP clinical trials. The test-retest variability for three different sensitivity indices, individual test loci sensitivity, mean sensitivity, and volume sensitivity, was identified to define the requirement for a clinically significant change after accounting for natural variability. The appropriateness of using the 5 points with 7.0 or more dB pointwise change as a microperimetry endpoint was explored, alongside the variability of the three sensitivity indices over time.

## Methods

Patients with *RPGR*-associated RP attended Oxford Eye Hospital, a tertiary referral centre for inherited retinal degeneration. Repeatability data were collated retrospectively from clinical investigations undertaken as part of the eligibility screening process but before the recruitment into gene therapy clinical trials (UK research ethics committee reference: 16/SC/0551). Longitudinal data were collated retrospectively from microperimetry tests undertaken as part of the Visual Function in Retinal Degeneration study (UK research ethics committee reference: 20/WM/0283) and from patients undergoing routine clinical care. All patients had a confirmed pathogenic mutation in the *RPGR* gene to accompany their clinical diagnosis of X-linked RP. Patients with co-pathologies were excluded from the study.

### Microperimetry Assessment

Central retinal sensitivity assessment using the MAIA microperimeter was performed on all participants, in a darkened room (light level <1.0 lux). The standard 10-2 test grid was used, with 4-2 bracketing threshold strategy. Examination involved the presentation of Goldmann size III stimuli of various intensities (0–318 cd/m^2^), presented for 200 ms, on to a mesopic background (1.27 cd/m^2^). The overall dynamic testing range was 0 to 36 dB. A red circle with a diameter of 1° was used as a central fixation target. Before testing, subjects were given verbal test instructions. The right eye was tested first consistently, followed by the left eye as per clinical convention. The nontested eye was occluded throughout. All tests were completed by trained optometrists who were certified as part of the gene therapy clinical trials.

The repeatability arm completed repeat (triplicate) microperimetry testing across two consecutive days. The first test was a ‘new expert examination’ with all following examinations (test two and test three) conducted using the ‘follow-up’ functionality on the MAIA machine, ensuring alignment of the grid testing locations between all three examinations. Microperimetry was performed after 20 minutes’ dark adaption without pupil dilation. Examinations were judged as reliable based on the response frequency to a 10-dB stimuli presented to the physiological blind spot approximately once every minute, termed ‘fixation losses’. Owing to the eye-tracking capability of the MAIA, these responses are commonly considered to be false positives arising from incorrect button presses in the absence of any seen stimuli. Any examinations with fixation losses of 30% or greater were deemed to be unreliable and were repeated.

The longitudinal arm completed microperimetry assessments under the same testing conditions by the same optometrists but without any formal dark adaptation or pupil dilation.^13^^,^^14^ Microperimetry tests were completed at visit one and repeated at their next clinical follow-up visit (visit two) on both eyes, without any formal learning examination. Similarly, any tests with fixation losses of 30% or greater were deemed to be unreliable and not included in the analyses.

### Statistical Analyses

Three retinal sensitivity indices were analyzed: pointwise sensitivity, mean sensitivity, and volume sensitivity. Pointwise sensitivity and mean sensitivity form part of the standard MAIA output, measured in decibels dB. Volume sensitivity, measured in decibel*degrees squared (dB*deg^2^), was calculated for each patient examination using the freely available open source MAIA3D application (https://ocular.shinyapps.io/MAIA3D).^7^

For the triplicate testing, test one was considered a learning test and not considered further, following findings from a previous study reporting increased test–retest variability between test one and test two.^15^ Test two and test three results were used for Bland–Altman repeatability analyses.^16^ The coefficient of repeatability (CoR) was used to define the requirement for a clinically significant change that is beyond natural variability. Linear mixed modelling was used to account for repeated measures (68 loci tested per patient) with each patient fitted with a random intercept. Stimulus location was set as the fixed effect independent variable and pointwise sensitivity value as the dependent response variable. Mixed modelling treatments, where many loci are tested per patient or where more than two examinations are compared per patient, are often neglected. However, this is paramount to avoiding bias due to potential clustering of repeated measures (68 loci) from each patient. The parametric independent method of bootstrapping with a resampling rate of 1000 was used to estimate population means and confidence intervals within these skewed or zero-inflated data samples. R code for generating Bland–Altman statistics and plots with options for repeated measures and bootstrapping have been made freely available as the R package “blandultim” and can be accessed from https://github.com/amanasj.

Non-parametric descriptive statistical analyses were applied accordingly using SPSS (version 27; IBM, Armonk, New York, NY), including medians and interquartile ranges (IQR). Statistical significance was set at a *P* value of less than or equal to 0.05. Further figures were created using GraphPad Prism (version 9.4.1; GraphPad Software, San Diego, CA).

## Results

### Repeatability Analysis in *RPGR*-associated RP

Fourteen participants with *RPGR*-associated RP completed triplicate microperimetry testing. One patient was subsequently excluded from further analysis because they showed no detectable central retinal sensitivity at any loci using the MAIA device. The remaining 13 patients (median age, 27 years; IQR, 24–38 years) had moderately well preserved VA indicated with the Early Treatment Diabetic Retinopathy Study (ETDRS) chart, median 74 ETDRS letters (IQR, 64–75 ETDRS letters) and 74 ETDRS letters (IQR, 68–77 ETDRS letters) for the right and left eyes, respectively. Test two median test duration was 6:56 minutes (IQR, 5:27–7.28 minutes) and 7:09 minutes (IQR, 5.24–8.23 minutes) for the right and left eyes, respectively. Test three median duration was 6:11 minutes (IQR, 5:36–7:55 minutes) and 5:58 minutes (IQR, 4.21–7:14 minutes) for the right and left eyes, respectively.

The RPGR-associated RP repeatability data showed three test pairs from three different participants with a 7.0 dB or greater sensitivity change in five or more loci. This included one participant who met the criteria showing a 7.0 dB or greater gain in five loci between test two and test three. Another participant showed a 7.0 dB or greater decline in five loci, while a third participant showed a mixed response with a 7.0 dB or greater gain in two loci and ≥7.0 dB decline in five loci. Thirteen test pairs demonstrated between one and four loci with 7.0 dB or greater mixed (gain and decline) change. Despite these remaining 13 test pairs not meeting the FDA criteria, of requiring 5 loci with 7.0 dB or greater gain, this finding still highlights the typical degree of natural variability encountered. Only eight test pairs showed no loci with a 7.0 dB or greater absolute change.

**Figure 1.**
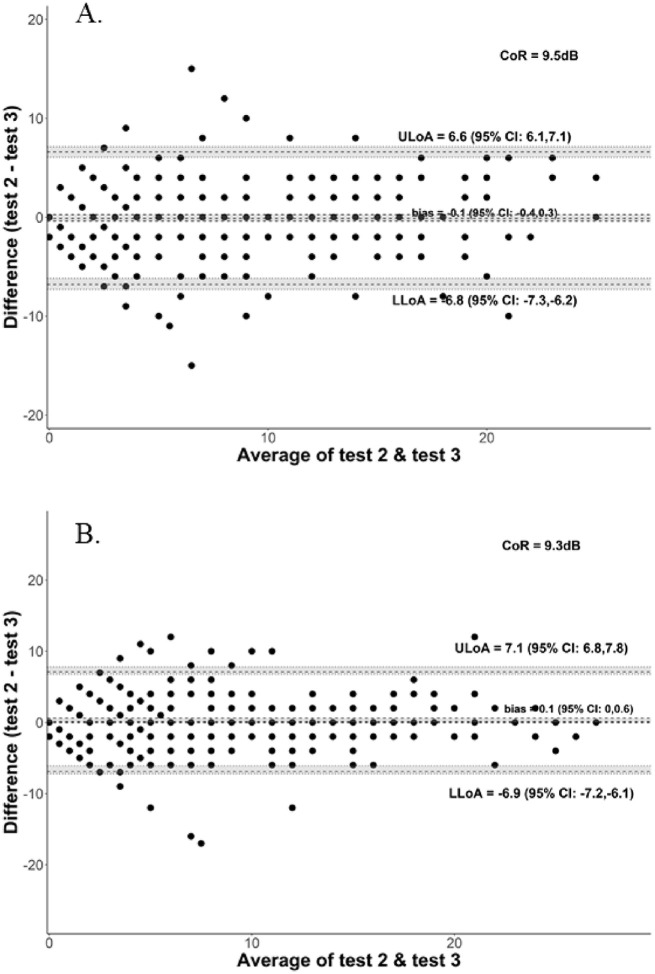
The pointwise repeatability for the right (A) and left eyes (B). The Bland–Altman plots illustrate the test–retest variability of individual microperimetry loci for participants with RPGR-associated RP. The pointwise CoR for the right and left eyes were ±9.5 dB and ±9.3 dB respectively. LLoA, lower limit of agreement; ULoA, upper limit of agreement.

The pointwise CoR (Fig. 1) was ±9.5 dB and ±9.3 dB, for the right and left eyes, respectively, rounded down to ±9.0 dB for convenience. This, by definition, means that any repeat test points should fall within ±9.0 dB of the first test result for that locus with a 95% probability. Therefore, there is a 5% probability a repeat pointwise test result will exceed ±9.0 dB of the first test for that locus. Summing probabilities by applying a binomial cumulative distribution function results in an overall 25% probability of obtaining five or more points (out of the 68 tested) with a greater than ±9.0 dB change from baseline due to variability alone. The probability of achieving 5 points with the less stringent criteria of ±7.0 dB would be expected to be even greater than 25%. However, if only the central 16 points were considered, then the probability of obtaining 5 points with a change of greater than ±9.0 dB reduces to a more reasonable 0.1%. For 36 central points, the probability of achieving 5 points with a change of greater than ±9.0 dB would be approximately 3%.

For the mean sensitivity, the CoR was much lower (±0.7 dB and ±1.3 dB in the right and left eyes, respectively) than the pointwise sensitivity CoR because it reflects an averaged global measure, which is less affected by individual point changes. The volume sensitivity CoR was ±144.5 dB*deg^2^ and ±324.2 dB*deg^2^, for the right and left eyes, respectively. Both the CoR for mean sensitivity and volume sensitivity showed greater variability in the left eye than the right eye; however, there was no statistically significant difference between test two minus test three, for each eye, for each sensitivity index (Wilcoxon signed-rank test; adjusted *P* > 0.05).

### Longitudinal Central Retinal Sensitivity Changes Between Visit One and Visit Two

Fourteen participants (median age, 23 years; IQR, 17–33 years), with *RPGR*-associated-RP completed a single microperimetry examination on each eye on two separate visits. The median follow-up interval was 12 months (IQR, 4–18 months). Fifty-six microperimetry examinations were completed in total. Two examinations from two different participants (one right eye examination and one left eye examination) had fixation losses of more than 30% and so were excluded from further analyses; however, the corresponding fellow eye examinations, which had 30% or less fixation losses were included. Therefore, 13 test pairs (visit one and visit two) were analyzed from 14 participants, forming the longitudinal dataset. Median VA was 65 ETDRS letters (IQR, 56–80 ETDRS letters) and 66 ETDRS letters (IQR, 63–72 ETDRS letters), for the right and left eyes, respectively.

**Figure 2.**
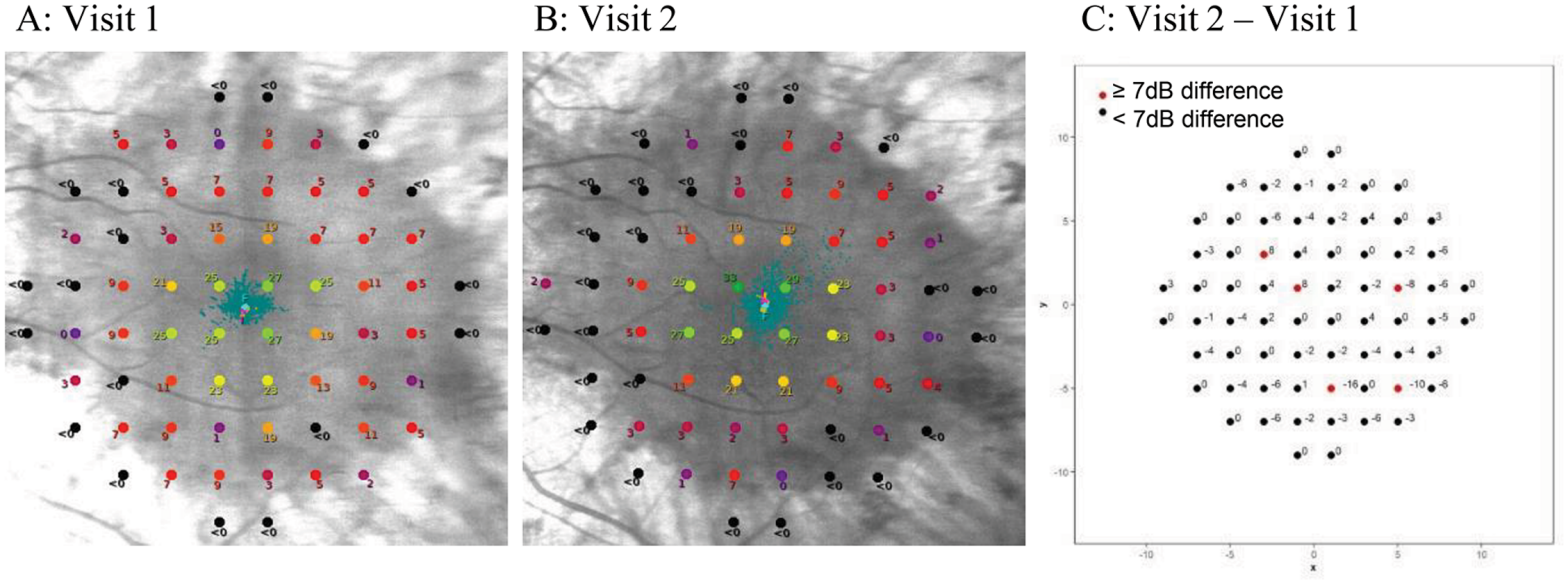
Longitudinal pointwise sensitivity changes for a single test pair: (A) and (B) show MAIA pointwise sensitivity threshold plots from a single participant, for visit one and two, respectively. (C) Details visit two minus visit one pointwise sensitivity differences. The red points highlight any loci with absolute changes of ≥7.0 dB, in this instance two loci that gained ≥7.0 dB and three loci dropped <7.0 dB.

Individual point sensitivity analyses of the longitudinal data, using 5 points with a sensitivity change of equal to or more than 7.0 dB as a clinically significant cut-off (Fig. 2), showed two test pairs with a clinically significant gain. One of these gains was also within the central 16 loci. To reiterate, these gains are from natural variability alone with no clinical intervention. The other test pair showing a gain, also showed a 7.0 dB or greater decline in at least 5 loci indicating mixed variability. Overall, 14 test pairs showed 7 dB or greater decline in 5 or more loci (Table). Nine test pairs showed mixed responses where some loci gained and others declined by 7 dB or more sensitivity, in cumulatively 5 or more loci, indicating further variability, while a further six test pairs showed between one to four loci with a 7 dB or greater sensitivity change. There were no test pairs that showed any loci with no significant change. Since *RPGR*-associated RP is a progressive condition, we would expect a certain level of microperimetry sensitivity decline; however, any gains are more likely owing to variability.

**Table.** Number of Test Pairs From the Longitudinal Data Showing a Clinically Significant Sensitivity Change

	No. of Test Pairs (*n* = 26)		
	PTW Sensitivity Change (≥7 dB in ≥5 Loci)	MS Change (≥1.3 dB)	Volume Change (≥324.2 dB*deg^2^)
Clinically significant gain	2^a^	0	0
Clinically significant decline	14^b^	12	10

a One test pair showed both a significant pointwise gain and decline

b MS, mean sensitivity; PTW, pointwise;.

In comparison, global sensitivity indices, using the CoR for mean sensitivity (±1.3 dB, left eye CoR) and volume sensitivity (±324.2 dB*deg^2^, left eye CoR), showed no test pairs with clinically significant gains between the two visits. Instead, more than one-half of the test pairs showed sensitivities within natural variability (CoR), which is expected given the short follow-up interval. The remaining test pairs demonstrated clinically significant declines (Table).

The concordance of clinical significance across the three sensitivity indices showed four test pairs with a significant decline and two a significant gain (including the one test pair that showed mixed significant changes) based on pointwise sensitivity cut-offs (≥7 dB in ≥5 loci), despite within test–retest variability mean and volume sensitivity changes (Supplementary Table S1). Meanwhile, two test pairs showed significant mean sensitivity declines (≥1.3 dB), but showed nonsignificant pointwise and volume sensitivity changes. The remaining 18 of 26 test pairs (69%) showed agreement in sensitivity change categorization across the three indices.

The overall longitudinal microperimetry median mean sensitivity difference between visit one and visit two was −0.2 dB (IQR, −2.2 to 0.0 dB) for the right eye and −1.40 dB (IQR, −2.8 to 0.7 dB) for the left eye. The left eye sensitivity decline was slightly above the test–retest variability (CoR: ±1.3 dB, as reported elsewhere in this article), suggesting a clinically significant loss beyond natural variability, which was also statistically significant (Wilcoxon signed rank test; adjusted *P* = 0.006). The right eye, however, demonstrated no clinical or statistical significant sensitivity change (Wilcoxon signed rank test; adjusted *P* > 0.05).

In comparison, the median volume sensitivity changes showed no clinically significant change (>324.2 dB*deg^2^, as reported elsewhere in this article), beyond natural variability, for the right and left eyes (−83.4 dB*deg^2^ [IQR, −508.3 to 18.2 dB*deg^2^] and −314.6 dB*deg^2^ [IQR, −674.0 to 143.3 dB*deg^2^], respectively). Like the mean sensitivity, the left eyes showed a statistically significant decline in volume sensitivity between visits one and two (Wilcoxon signed rank test, adjusted *P* = 0.004).

### Impacts of Multiple Scotoma Points

**Figure 3.**
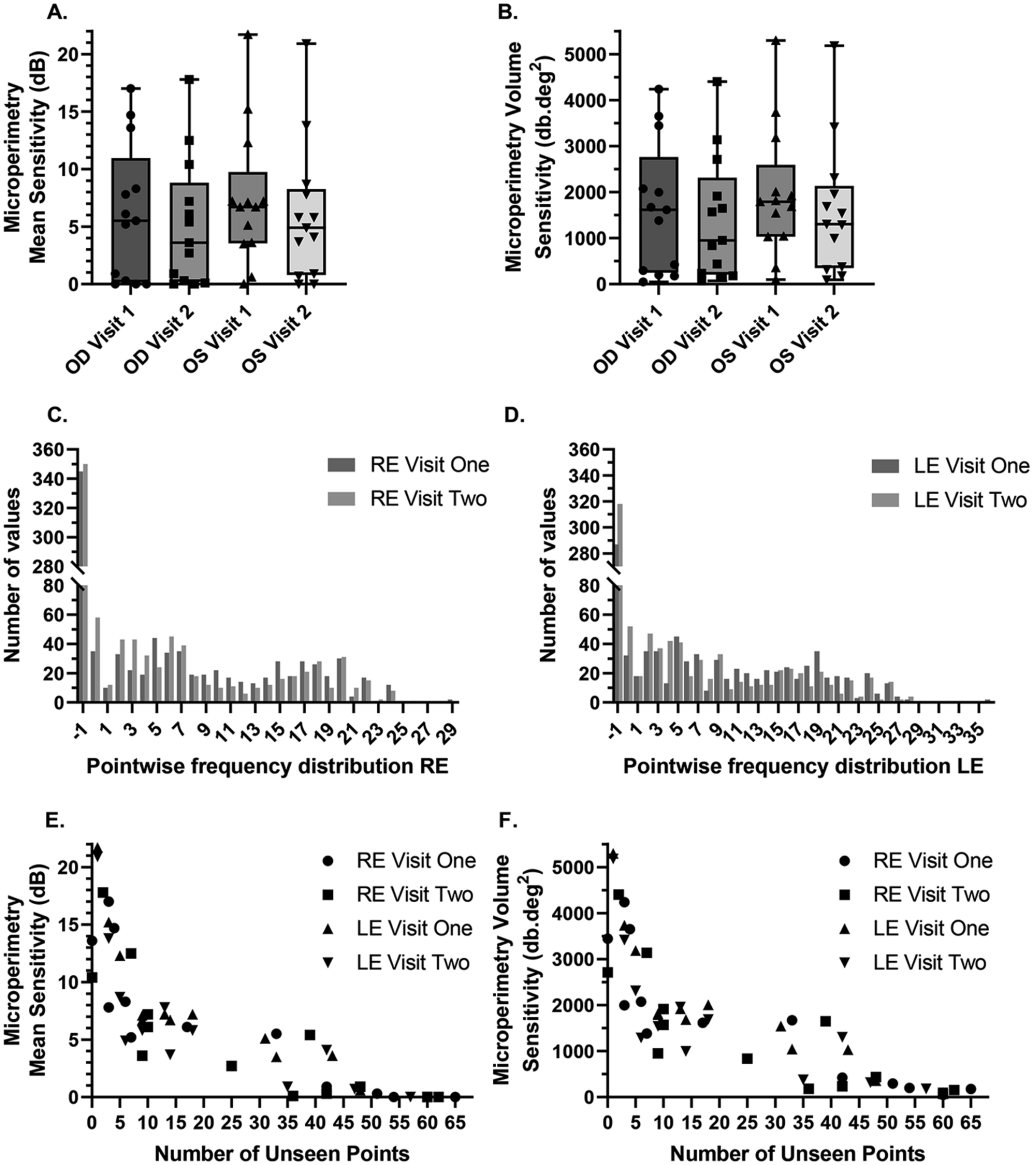
(A) The median mean sensitivity for the right eyes visit one was 5.7 dB (IQR, 0.2–9.2 dB) and for visit two it was 3.2 dB (IQR, 0.3–8.5 dB). Left eye median mean sensitivity for visit one was 6.9 dB (IQR, 2.8–9.2 dB), for visit two it was 4.3 dB (IQR, 0.5–9.3 dB). (B) The volume sensitivity data for visits one and two for each eye. The median volume sensitivity for the right eye was 1497.4 dB*deg^2^ (IQR, 272.3–2460.2 dB*deg^2^) for test one and 893.7 dB*deg^2^ (IQR, 223.3–2217.8 dB*deg^2^) for test two. Left eye median volume sensitivity was 1748.2 dB*deg^2^ (IQR, 860.9–2435.3 dB*deg^2^) for visit one and 1143.1 dB*deg^2^ (IQR, 284.8–2322.0 dB*deg^2^) for visit two. (C and D) Frequency distribution plots for microperimetry pointwise sensitivity values, from visit one and two, showing a non-normal positively skewed distribution, with −1.0 dB containing the highest number of values for the right and left eyes, respectively. (E and F) Scatter plots showing the relationship between the number of unseen points and sensitivity, for mean sensitivity and volume sensitivity, respectively. Right eye (RE); left eye (LE).

In *RPGR-*associated RP, many patients have a parafoveal circular ring scotoma that progresses radially inward and outward over time. As the area of central retinal sensitivity constricts, a number of nonseen loci around the parafoveal tested area arise. In total, 884 pointwise loci were included as part of the longitudinal dataset, for each eye across all visits. The percentages of nonseen stimuli were between 32% and 40% for visits one and two, for both eyes. This reflects the significant positive skew of the pointwise sensitivity frequency distribution evident in Figures 3C and 3D. When more than one-half of the number of test loci (>34) have no detectable sensitivity and are assigned −1.0 dB, the overall mean sensitivity becomes very low (<1.0 dB). Once the number of nonseen loci extends to 50 or more points (out of a total of 68 points), the mean sensitivity is driven down to the floor of zero (Fig. 3E).

From the longitudinal data, five right eye and three left eye examinations had very low mean sensitivity values (<1.0 dB) owing to the domination of these scotomatous regions. Five right eye tests and three left eye tests, from three different participants, showed no detectable mean sensitivity (0.0 dB). Despite this finding, all participants had measurable sensitivity at several individual loci. In these individuals median sensitivity of only seen points was 3.1 dB (IQR, 2.8–4.35 dB).

With volume sensitivity, all tests showed measurable volumetric sensitivity, with a greater dispersion of values in the lower ranges of sensitivity corresponding to those with late disease stages (see plot tails in Fig. 3F) compared with mean sensitivity (Fig. 3E). As the number of nonseen points increased, volume sensitivity was still able to capture sensitivity values; unlike the mean sensitivity index, volume sensitivity was less hindered by floor effects. This is a result of the nonseen stimuli (−1.0 dB) being reassigned to 0.0 dB and just seen at the brightest setting (0.0 dB) being reassigned with the nominal value of 0.1 dB, as used in static automated perimetry.^8^ Volumetric measures will only return a value of zero when all points are truly not seen.

## Discussion

Microperimetry pointwise sensitivities show significantly greater variability than global sensitivity measures, such as mean sensitivity and volume sensitivity indices. As such, the five-point 7.0 dB or greater sensitivity gain, as a microperimetry endpoint is more prone to detecting sensitivity changes due to increased natural variability alone. Conversely, mean sensitivity indices are less prone to variability (owing to smoothing over all points); however, they do not accurately represent the overall sensitivity results in those with a skewed distribution of sensitivities. Since volume sensitivity is a product of total sensitivity and spatial information, it is more sensitive to small, localized changes and is unaffected by averaging effects. Volume sensitivity may be more useful for assessing patients with low levels of central retinal sensitivity.

The CoR for each sensitivity index can be used to inform what a clinically significant sensitivity change is required after accounting for natural variability. Clinical significance is different from statistical significance. A comparison of test results can be statistically significantly different; however, this difference is meaningless if these differences are within natural test–retest variation (i.e., not clinically significant). The pointwise sensitivity CoR values compare well to previously reported values for choroideremia (±8.7 dB), where it was considered later stage patients show greater variability.^17^ In cases where treatment may be applied in patients with drastically different stages of disease, a single criterion is unlikely to be appropriate across the full spectrum of disease. Instead, individual measures of clinical significance may aid the detection of meaningful treatment effects. Further investigation is required to understand how much test–retest repeatability varies across different disease stages. To the best of our knowledge, this work is the first time CoR values have been reported for microperimetry volume sensitivity in patients with *RPGR*-associated RP.

Retinal physiology differs significantly from the cone rich macula, where cones connect directly with a single ganglion cell, to the rod rich peripheral retina, where multiple photoreceptors summate and feed into a ganglion cell. Standard microperimetry assesses the macula region only and so is believed to represent predominantly cone photoreceptor function.^13^ Wider field standard automated perimetry is more representative of peripheral ganglion cell function.^20^ Therefore, in standard automated perimetry, subtle variation in retinal testing location is unlikely to have a significant impact on the pointwise sensitivity in the periphery, unlike in microperimetry. Furthermore, significant differences in test parameters such as background luminance, stimulus dynamic ranges, stimulus size, and absolute light intensity intervals exist between the two testing modalities. As a result, a 7.0-dB change within microperimetry is unlikely to be clinically equivalent to a 7.0-dB change in standard automated perimetry. Although it can be useful to draw on previous visual field literature, caution should be exercised in using clinical trial end points intended for standard automated perimetry (with different study designs) for studies involving microperimetry.

The limited raster refresh rate (25 Hz) of the infrared scanning laser ophthalmoscope fundus tracker, on the MAIA microperimeter, could be one source of pointwise variability.^7^ Rapid eye movements occurring between each frame may go undetected and result in stimuli positioning errors and, therefore, less correspondence in retinal location testing.^18^ This difference is exacerbated at transition zones (regions between healthy and degenerated retina), which have shown greater levels of variability.^19^ Another source of potential variability may arise from the initial fundus imaging setup. In poorly focused images, co-registration of the fundus landmarks in subsequent examinations can limit intertest alignment accuracy.^7^

A further pointwise sensitivity limitation arises in subjects with no detectable sensitivity (corresponding with non-seen stimuli) at multiple loci, since the pointwise sensitivity is arbitrarily assigned a value of −1.0 dB by MAIA, reflecting the floor effect of the machines’ capabilities. If the same locus, at a subsequent visit, shows a 6.0-dB sensitivity value, it is unknown whether this change should constitute a gain of 7.0 dB or greater, as the exact baseline sensitivity value is unknown. In this study, a −1.0 dB to 6.0 dB change between visit one and visit two was classified as a gain of 7.0 dB.

Using clustered pointwise sensitivity endpoints, that are focused on a specific area of anticipated treatment effects, may minimize the impacts of individual natural loci variability and reduce the probability of false positives (patients meeting the endpoint due to natural variability). The probability of 3.0% or 0.1% false-positive rate achieved by only considering pointwise changes within the central 36 or central 16 points, respectively, could be more amenable. However, since many patients with *RPGR*-associated RP have preserved central sensitivity, the potential for detecting therapeutic sensitivity gains within the central 16 or 36 points remains unknown. Furthermore, sensitivity changes occurring outside the clustered analysis predefined area will be missed.

Further sources of variability include fatigue. It is convention to test the right eye first, followed by the left eye, and microperimetry testing typically exceeds 5 minutes testing time per examination per eye. The greater CoR seen in the left eyes for both mean and volume sensitivity suggests fatigue effects could be a large source of variability, especially since disease progression has been shown to be highly symmetrical between eyes in patients with *RPGR*-associated RP.^20^ In a clinical trial setting, it is important to minimize sources of variability to improve detection of treatment related gains. To date, *RPGR*-associated RP clinical trial design has used a single study eye (usually the worse eye) and used the nontreated eye as an internal control.^11^^,^^12^ Variability arising from fatigue could be reduced if only the study eye is tested and a separate cohort is used as a control; however, this strategy would reduce potential sample sizes, which, in rare diseases, may not be feasible. Furthermore, careful consideration of visual function testing order may minimize variability due to fatigue effects; if microperimetry was selected as the primary outcome measure, perhaps this should be completed prior to any other visual function testing, to minimize fatigue.

Mean sensitivity is currently the standard microperimetry index, it is easy to understand and readily available on MAIA outputs. Testing grids with uniform stimuli spacing (such as the 10-2 grid used in this study) was previously considered favorable in the context of mean sensitivity calculations.^7^ However, this study demonstrates, in those with very constricted fields and many nonseen stimuli, the averaging effects of very skewed data, driving the mean sensitivity toward zero. Subsequently, this factor reduces the visual function information gained and could lead to exclusion of patients with late-stage disease (with a mean sensitivity of 0 dB) from clinical trial enrolment, since percentage change statistical analyses cannot be performed. Furthermore, it may limit the potential to detect therapeutic changes in patients with scotoma areas greater than the residual functioning area. A modified mean sensitivity approach could include seen stimuli only; however, this strategy introduces the opposite problem of biasing the mean toward the greater sensitivity values.

The study contains several limitations. There was no formal microperimetry learning test undertaken in the longitudinal testing. Performing a microperimetry learning test before examination has previously been recommended.^15^ However, another study reported a high interclass correlation coefficient for microperimetry testing in patients with *RPGR*-associated RP who completed repeat microperimetry tests, without any prior learning tests.^21^ Furthermore, in clinical trials, although it may be conventional to undertake repeat or triplicate baseline examinations, subsequent follow-up visits do not include learning tests. The duration of learning effects is unknown and so it is unclear whether a learning test is necessary at every follow-up visit, particularly where a substantial amount of time (e.g., 6 or 12 months) has passed since initial testing. The higher left eye CoR for both mean and volume sensitivity suggests that fatigue effects were more problematic than learning effects, since it is convention to test the left eye after the right eye. Using the higher CoR for the clinically significant cut-off, in the longitudinal analyses, could be considered overly conservative. A lower CoR cut-off is likely to increase the number of individuals identified as having clinically significant changes between visits. The study is also limited by the small sample size; however, due to the rarity of *RPGR*-associated RP it is difficult to recruit large numbers of patients, and *RPGR*-associated RP clinical trials are likely to reflect similarly small sample sizes.

## Conclusions

Clinical trials should report the test–retest variability, in the sensitivity index being used, for the population being studied, to determine the criteria of a clinically significant change. This factor is crucial to ascertain before drawing conclusions on possible treatment effects. Pointwise sensitivity indices should be used with caution as outcome measures in clinical trials due to high levels of test–retest variability. Global indices appear less prone to variability. Volume sensitivity indices may be superior for use in *RPGR-*associated RP clinical trials compared with mean sensitivity because they are not affected by averaging effects. Further investigation using longer follow-up data is required to assess the sensitivity of volume indices to detect disease progression and correlation with structural markers in RP and beyond.

## Supplementary Material

Supplement 1

## References

[1] Sharon D, Sandberg MA, Rabe VW, et al. RP2 and RPGR mutations and clinical correlations in patients with X-linked retinitis pigmentosa. *Am J Hum Genet*. 2003; 73(5): 1131–1146. 14564670 10.1086/379379 PMC1180492

[2] Cehajic-Kapetanovic J, Xue K, Martinez-Fernandez de la Camara C, et al. Initial results from a first-in-human gene therapy trial on X-linked retinitis pigmentosa caused by mutations in RPGR. *Nat Med*. 2020; 26(3): 354–359. 32094925 10.1038/s41591-020-0763-1 PMC7104347

[3] Georgiou M, Awadh Hashem S, Daich Varela M, et al. Gene therapy in X-linked retinitis pigmentosa due to defects in RPGR. *Int Ophthalmol Clin*. 2021; 61(4): 97–108. 34584047 10.1097/IIO.0000000000000384

[4] Di Iorio V, Karali M, Melillo P, et al. Spectrum of disease severity in patients with X-linked retinitis pigmentosa due to RPGR mutations. *Invest Ophthalmol Vis Sci*. 2020; 61(14): 36. 10.1167/iovs.61.14.36 PMC7774109 33372982

[5] Buckley TMW, Jolly JK, Josan AS, et al. Clinical applications of microperimetry in RPGR-related retinitis pigmentosa: a review. *Acta Ophthalmol*. 2021; 99(8): 819–825. 33783139 10.1111/aos.14816

[6] Yang Y, Dunbar H. Clinical perspectives and trends: microperimetry as a trial endpoint in retinal disease. *Ophthalmologica*. 2021; 244(5): 418–450. 33567434 10.1159/000515148 PMC8686703

[7] Pfau M, Jolly JK, Wu Z, et al. Fundus-controlled perimetry (microperimetry): Application as outcome measure in clinical trials. *Prog Retin Eye Res*. 2021; 82: 100907. 33022378 10.1016/j.preteyeres.2020.100907 PMC12872260

[8] Josan AS, Buckley TMW, Wood LJ, et al. microperimetry hill of vision and volumetric measures of retinal sensitivity. *Transl Vis Sci Technol*. 2021; 10(7): 12. 10.1167/tvst.10.7.12 PMC8196404 34110386

[9] Weleber RG, Smith TB, Peters D, et al. VFMA: topographic analysis of sensitivity data from full-field static perimetry. *Transl Vis Sci Technol*. 2015; 4(2): 14. 10.1167/tvst.4.2.14 PMC4413926 25938002

[10] Weinreb RN, Kaufman PL. The glaucoma research community and FDA look to the future: a report from the NEI/FDA CDER Glaucoma Clinical Trial Design and Endpoints Symposium. *Invest Ophthalmol Vis Sci*. 2009; 50(4): 1497–505. 19321793 10.1167/iovs.08-2843

[11] Yang P, Lauer A, Pennesi ME, et al. Six month findings from a phase 1/2 clinical study of subretinal gene therapy drug AGTC-501 for X-linked retinitis pigmentosa show clinically meaningful improvement in macular sensitivity. *Invest Ophthalmol Vis Sci*. 2021; 62(8): 1481.

[12] von Krusenstiern L, Liu J, Liao E, et al. Changes in retinal sensitivity associated with cotoretigene toliparvovec in X-linked retinitis pigmentosa with rpgr gene variations. *JAMA Ophthalmol*. 2023; 141(3): 293. 36757689 10.1001/jamaophthalmol.2022.6254 PMC9912164

[13] Han RC, Gray JM, Han J, et al. Optimisation of dark adaptation time required for mesopic microperimetry. *Br J Ophthalmol*. 2019; 103(8): 1092–1098. 30269100 10.1136/bjophthalmol-2018-312253

[14] Han RC, Jolly JK, Xue K, et al. Effects of pupil dilation on MAIA microperimetry. *Clin Exp Ophthalmol*. 2017; 45(5): 489–495. 28002873 10.1111/ceo.12907

[15] Buckley TMW, Jolly JK, Menghini M, et al. Test-retest repeatability of microperimetry in patients with retinitis pigmentosa caused by mutations in RPGR. *Clin Exp Ophthalmol*. 2020; 48(5): 714. 32220136 10.1111/ceo.13753

[16] Bland MJ, Altman D. Statistical methods for assessing agreement between two methods of clinical measurement. *Lancet*. 1986; 327(8476): 307–310. 2868172

[17] Jolly JK, Xue K, Edwards TL, et al. Characterizing the natural history of visual function in choroideremia using microperimetry and multimodal retinal imaging. *Invest Ophthalmol Vis Sci*. 2017; 58(12): 5575–5583. 29084330 10.1167/iovs.17-22486 PMC5850987

[18] Wu Z, Jung CJ, Ayton LN, et al. Test-retest repeatability of microperimetry at the border of deep scotomas. *Invest Ophthalmol Vis Sci*. 2015; 56(4): 2606–2611. 25813990 10.1167/iovs.14-15977

[19] Hood DC, Lazow MA, Locke KG, et al. The transition zone between healthy and diseased retina in patients with retinitis pigmentosa. *Invest Ophthalmol Vis Sci*. 2011; 52(1): 101–108. 20720228 10.1167/iovs.10-5799 PMC3053270

[20] Harwerth RS, Carter-Dawson L, Shen F, et al. Ganglion cell losses underlying visual field defects from experimental glaucoma. *Invest Ophthalmol Vis Sci*. 1999; 40(10): 2242–2250. 10476789

[21] Anikina E, Georgiou M, Tee J, et al. Characterization of retinal function using microperimetry-derived metrics in both adults and children with RPGR-associated retinopathy. *Am J Ophthalmol*. 2022; 234: 81–90. 34303686 10.1016/j.ajo.2021.07.018 PMC8847997

