# Supplementary material for: Microperimetry as an Outcome Measure in RPGR-associated Retinitis Pigmentosa Clinical Trials
Source: Transl Vis Sci Technol. 2023 Jun 9;12(6):4. doi: 10.1167/tvst.12.6.4 (PMC10259674; doi:10.1167/tvst.12.6.4)
Supplement: Supplement 1 [file tvst-12-6-4_s001.pdf]

Supplementary Material

Table S1: Categorization of test pair sensitivity change between visit one and two (longitudinal data) for each of the three sensitivity indices.

**ID**<br>**OD**	**Significant PWS change (≥7dB in ≥5 loci)**	**Significant MS change (≥1.3dB)**	**Significant VS change (≥324.2dB*deg2)**
**1**	Significant Decline	Significant Decline	Significant Decline
**2**	Significant Decline	Significant Decline	Significant Decline
**3**	Significant Decline	Significant Decline	Significant Decline
**4**	Within Test Retest	Within Test Retest	Within Test Retest
**5**	Significant Decline	Within Test Retest	Within Test Retest
**6**	Significant Decline	Significant Decline	Significant Decline
**7**	Within Test Retest	Within Test Retest	Within Test Retest
**8**	Within Test Retest	Within Test Retest	Within Test Retest
**9**	Within Test Retest	Within Test Retest	Within Test Retest
**10**	Within Test Retest	Within Test Retest	Within Test Retest
**11**	Within Test Retest	Within Test Retest	Within Test Retest
**12**	Significant Decline	Significant Decline	Significant Decline
**13**	Significant Gain	Within Test Retest	Within Test Retest
**OS**			
**1**	Significant Decline	Significant Decline	Significant Decline
**2**	Significant Decline	Significant Decline	Significant Decline
**3**	Significant Decline	Significant Decline	Significant Decline
**4**	Within Test Retest	Within Test Retest	Within Test Retest
**5**	Significant Gain & Decline	Within Test Retest	Within Test Retest
**6**	Within Test Retest	Significant Decline	Within Test Retest
**7**	Significant Decline	Significant Decline	Significant Decline
**8**	Significant Decline	Significant Decline	Significant Decline
**9**	Within Test Retest	Within Test Retest	Within Test Retest
**11**	Significant Decline	Within Test Retest	Within Test Retest
**12**	Within Test Retest	Within Test Retest	Within Test Retest
**13**	Significant Decline	Within Test Retest	Within Test Retest
**14**	Within Test Retest	Significant Decline	Within Test Retest

*PWS pointwise sensitivity, MS mean sensitivity, VS volume sensitivity
